# Understanding the role of membrane cholesterol upon Epstein Barr virus infection in astroglial cells

**DOI:** 10.3389/fimmu.2023.1192032

**Published:** 2023-10-09

**Authors:** Annu Rani, Manushree Tanwar, Tarun Prakash Verma, Priyanka Patra, Pankaj Trivedi, Rajesh Kumar, Hem Chandra Jha

**Affiliations:** ^1^ Department of Biosciences and Biomedical Engineering, Indian Institute of Technology, Indore, India; ^2^ Materials and Device Laboratory, Department of Physics, Indian Institute of Technology, Indore, India; ^3^ Department of Chemistry, University of Pennsylvania, Philadelphia, PA, United States; ^4^ Department of Experimental Medicine, Sapienza University of Rome, Rome, Italy

**Keywords:** Epstein Barr virus, multiple sclerosis, neurological diseases, cholesterol, therapeutics, Raman spectroscopy

## Abstract

**Background:**

EBV infection has long been postulated to trigger multiple sclerosis (MS) and anti-EBV antibodies showed a consistent presence in MS patients. Previous reports from our group have shown that the EBV infects different brain cells. Entry of the virus in neuronal cells is assisted by several host factors including membrane cholesterol. By using an inhibitor, methyl-β-cyclodextrin (MβCD), we evaluated the role of membrane cholesterol in EBV infection and pathogenesis

**Methodology:**

The membrane cholesterol depleted cells were infected with EBV and its latent genes expression were assessed. Further, EBV-mediated downstream signalling molecules namely STAT3, RIP, NF-kB and TNF-α levels was checked at protein level along with spatial (periphery and nucleus) and temporal changes in biomolecular fingerprints with Raman microspectroscopy (RS).

**Results:**

Upon treatment with MβCD, lmp1 and lmp2a suggested significant downregulation compared to EBV infection. Downstream molecules like STAT3 and RIP, exhibited a decrease in protein levels temporally upon exposure to MβCD while NF-kB levels were found to be increased. Further, the intensity of the Raman spectra exhibited an increase in triglycerides and fatty acids in the cytoplasm of EBV-infected LN-229 cells compared to MβCD+EBV. Likewise, the Raman peak width of cholesterol, lipid and fatty acids were found to be reduced in EBV-infected samples indicates elevation in the cholesterol specific moieties. In contrast, an opposite pattern was observed in the nucleus. Moreover, the ingenuity pathway analysis revealed protein molecules such as VLDLR, MBP and APP that are associated with altered profile of cholesterol, fatty acids and triglycerides with infection-related CNS disorders.

**Conclusion:**

Taken together, our results underline the important role of membrane cholesterol over EBV entry/pathogenesis in astroglia cells which further trigger/exacerbate virus-associated neuropathologies. These results likely to aid into the prognosis of neurological disease like MS.

## 1 Introduction

EBV is associated with various cancers and several neurological diseases like viral encephalitis, CNS-lymphoma, cerebral ataxia, meningitis, Multiple sclerosis (MS), Alzheimer’s disease (AD) and Parkinson’s disease (PD) (1, 2). Patients with history of infectious mononucleosis are reported to be more vulnerable to MS (3). Further, the cross-reactivity of antibodies was reported such as amino acids 411–440 of the viral protein EBV nuclear antigen 1 (EBNA1) with the human chloride-channel protein, anoctamin 2, α-crystallin B chain and glial cell adhesion protein (4). Notably, EBNA1 residues 411–426 suggested cross-reactivity with myelin basic protein (MBP) which is directly associated with MS (5, 6). Several anti-EBV drugs have been used as therapeutics for MS (7, 8). Therefore, these finding suggests that EBV is a vital factor in MS. Viruses and their associated factors acquire control over the brain cells and modulates neural niche. Membrane cholesterol facilitates the entry and assembly of various pathogens in the cells including viruses like EBV. Reports suggested that the entry of EBV and another gamma herpesvirus, KSHV, into epithelial cells is enabled by sphingolipids and cholesterol of the plasma membrane (9). These lipid assemblies are known to contain receptors which are tampered by the pathogens for their entry and/or egress (10, 11). Soon after infection, these pathogens tend to alter the process of lipid metabolism and contribute in neuropathologies (12, 13).

Membrane cholesterol-binding molecules like methyl-β-cyclodextrin (MβCD) and nystatin have been shown to reduce EBV infection in previous *in-vitro* studies (14, 15). On treatment with MβCD, the EBV latent membrane protein 2A (LMP2A), a crucial factor for viral latency and pathogenesis, has shown an increase in its exosome secretion (16). Besides, another virally encoded transmembrane protein, LMP1 also localizes in the cholesterol assemblies. It further activates a ligand-independent cascade through its two signalling domains namely C-terminal-activating regions 1 and 2 (CTAR1 and CTAR2), mimicking CD40 signalling (17). Subsequently, it activates the canonical nuclear factor kappa B (NF-kB), phosphoinositide-3-kinase-protein kinase B (PI3K-AKT) and epidermal growth factor receptor extracellular-regulated kinase-mitogen-activated protein kinase (ERK-MAPK) pathways (18, 19). The above-mentioned changes in the membrane cholesterol region-mediated signalling help the virus in its association and assembly (20). In addition, receptor-interacting protein (RIP) kinase interacts with TNF receptor-associated factor 2 (TRAF2) which is essential for the activation of NF-kB in a tumour necrosis factor-alpha (TNF-α) dependent manner (21). Viral infection results in the activation of various kinases leading to the phosphorylation of signal transducer and transcription 3 (STAT3) which ultimately migrates into the nucleus and regulate the expression of cytokines/chemokines (22). Furthermore, a plethora of tools has been used to unravel the altered biomolecular profile of host cells after infection with viruses and bacteria (23, 24). The Raman microspectroscopy (RS) is one such paramount non-destructive tool for quantitative and qualitative analysis of biomolecular fingerprints in cells, biofluids (i.e., serum) and tissues (11, 25). RS provides detailed information about the chemical structure, crystallinity and molecular interactions by interacting with chemical bonds in a given material (26).

Raman signals specific to biomolecules such as cholesterol, lipids, glucose, phenylalanine and phosphoinositide can be obtained from several pathogen-infected and uninfected samples (8). Significantly, alteration in the above-mentioned molecules is closely related to numerous neurological ailments (27, 28). As observed in the recent SARS-CoV-2 pandemic, viral infections and their outbreak are the results of rapid evolution. For limiting the virus-mediated spread, a holistic awareness of virus-mediated cell manipulations needs a better understanding (29). Although, numerous reports suggested the link of EBV with MS, yet no reports have highlighted the crucial role of membrane cholesterol. Therefore, the current study has taken on the challenge to elucidate the importance of membrane cholesterol upon EBV infection in astrocytes. Here, we showed for the first time the importance of membrane cholesterol in EBV-mediated downstream cascade with critical implications in causing neuroinflammation and associated disease pathologies. The transcript profile for EBV latent genes was investigated. In order to explore the downstream signalling pathway affected by EBV we have analysed the protein level of STAT3, RIP kinase, NF-kB and TNF-α. Lastly, RS was performed to explore the biomolecular profile of astroglia cells exposed to EBV in the presence and absence of the cholesterol inhibitor in a spatial and temporal-manner.

## 2 Materials and methods

### 2.1 Cell culture

The human glioblastoma cell LN-229 was acquired from Professor Kumaravel Somasundaram’s Lab, Department of Microbiology & Cell Biology, Indian Institute of Science Bangalore, India. For virus purification, HEK 293T cells were used which contain stably transfected bacterial artificial chromosome (BAC) green fluorescent protein (GFP)-EBV (30). The cells were cultured in Dulbecco’s modified Eagle’s medium (DMEM; Himedia Laboratories Pvt. Limited, India) supplemented with 10% fetal bovine serum (FBS; Himedia Laboratories Pvt. Limited, India), 50 U/ml, 100 μg/ml and 2 mM of penicillin, streptomycin and L-Glutamine respectively. The growing cell environment was humidified with 5% CO_2_ at 37°C. MβCD was obtained from Sigma-Aldrich Corp., St. Louis. MβCD was dissolved in milli-Q water and made the stock of 1mM.

### 2.2 Virus isolation and purification

The BAC-GFP-EBV was stably transfected into HEK 293T cells (31, 32), and were grown in complete DMEM with puromycin selection. EBV particles were obtained by using the protocol illustrated previously (33).

### 2.3 Cell cytotoxicity through MTT assay

For 3-(4,5-Dimethylthiazol-2-yl)-2,5-Diphenyltetrazolium Bromide (MTT) assay 10,000 LN-229 cells were seeded in each well of 96-well culture plate in complete DMEM supplemented with 10% of FBS and maintained for 24 hrs at 37°C with 5% CO_2_. MβCD was dissolved in milli-Q water and the final stock of 10 mM was prepared. Upon treatment with MβCD, the morphological changes in the cells were monitored using bright-field microscopy. After 24 hrs of treatment, medium was removed and 100 μL of fresh medium containing 0.5 mg/mL MTT was added to each well and incubated for 3 hrs at 37°C. MTT was removed and 100 μL of DMSO was added to dissolve formazan crystals by shaking for 2 hrs. Further, the absorbance was observed at 570 nm.

### 2.4 qRT-PCR

The EBV latent gene profile was analysed using qRT-PCR (**Table S1**). A total of 0.25 million cells were seeded in a 6-well plate and pre-treated with MβCD for 1 hr followed by EBV infection for 1, 2, 4, 6 and 12 hrs along with corresponding negative control. Total RNA extraction, complementary DNA preparation and qRT-PCR were carried out as described earlier (33). Gene-specific primers were designed from Primer-BLAST and are listed in **Table S1**. The qPCR was performed on two biological and two technical replicates with glyceraldehyde 3-phosphate dehydrogenase (GAPDH) as a housekeeping gene.

### 2.5 Western blotting

After exposure to MβCD and EBV for various time points, the LN-229 cells were harvested, washed with PBS and lysed in radioimmunoprecipitation assay buffer (RIPA) as described earlier (34). Antibodies against NF-kB (1:1000; D14E12 from Cell Signalling Technology (CST), Danvers, MA, USA), TNF-α (1:1000; D1G2 from CST), RIP (1:1000; D94C12 from CST), STAT3 (1;1000, 124H6 from CST) and GAPDH (1 μg/ml; AM4300; applied biosystems) were used for staining of the blots. Images of the blots were captured using the gel documentation system from BIO-RAD (ChemiDoc XRS+ System with Image Lab Software). Further, these images were analysed and quantified using Image J software (National Institutes of Health, Bethesda, MA, USA).

### 2.6 EBV infection and sample preparation for Raman microspectroscopy

LN-229 cells were grown to 40–60% confluency over coverslips in the six-well plate. Cells were gently washed with PBS and supplied with fresh media. The cells were then exposed to EBV. In MβCD-treatment, the cells were incubated with MβCD for 1 hr prior to the exposure to EBV. The uninfected control cells were harvested at 0 hr and fixed with 4% paraformaldehyde for 20 min at room temperature, washed with 1× PBS and stored at 4°C after drying. The other sets were separately harvested at 1, 2, 4, 6 and 12 hrs followed by fixing with PFA as mentioned before. The coverslips carrying fixed cells were arranged onto the glass slides such that cells face the upper side before visualisation under the microscope. Raman spectra was recorded from three different cells at three different points for each sample at nucleus and periphery (transmembrane and cytoplasmic conjunction region of the cells).

### 2.7 Raman microspectroscopy and spectral analysis

Raman microspectroscopy of the prepared samples was carried out using a LabRAM HR Evolution (Horiba-Jobin Yvon) spectrometer attached to an optical compound microscope. The excitation source was the He–Ne laser (λexc = 633 nm, ∼10 mW). The minimum possible laser power adjusted using a neutral-density filter was employed for the Raman measurement for subsiding the laser-induced biological sample damage. Spectral capturing of cells was done as mentioned previously (11). Further, the obtained spectra were analysed using OriginPro 2021. For the deconvolution of the Raman data, all the spectra were smoothed by 20 pts using the Savitzky–Golay filter in the signal processing to remove the irregularities and noise. The data was analysed by checking the changes in the Raman peak intensity, peak width and peak shift upon EBV and MβCD+EBV-infection. For intensity-based analysis, the average spectra obtained from the best three readings were considered for further analysis (10, 11). Peak width was checked using FWHM and the peak shifts were examined by comparing the wavenumber maxima difference of MβCD+EBV with EBV samples (35). Raman spectra shift was also calculated for MβCD+EBV samples after subtracting the wavenumber maxima with EBV-infected samples (**Table S3**).

### 2.8 Biomolecular connectome analysis

The ingenuity pathway analysis (QIAGEN) was performed for biomolecules acquired from the consecutive biomolecular change analysis in order to develop a connectome. The connectome was then percolated only for infectious diseases, inflammatory responses, and neurological disorders in the CNS and neuronal cell lines.

### 2.9 Statistical analysis

The t-test (Two-samples) was carried out to compare values of compound-treated samples with EBV infection samples. The t-statistic was significant at the 0.05 critical alpha level, P < 0.05 at the 95% confidence interval.

## 3 Results

### 3.1 Investigation of EBV latent genes after depleting the membrane cholesterol

**Figure 1:**
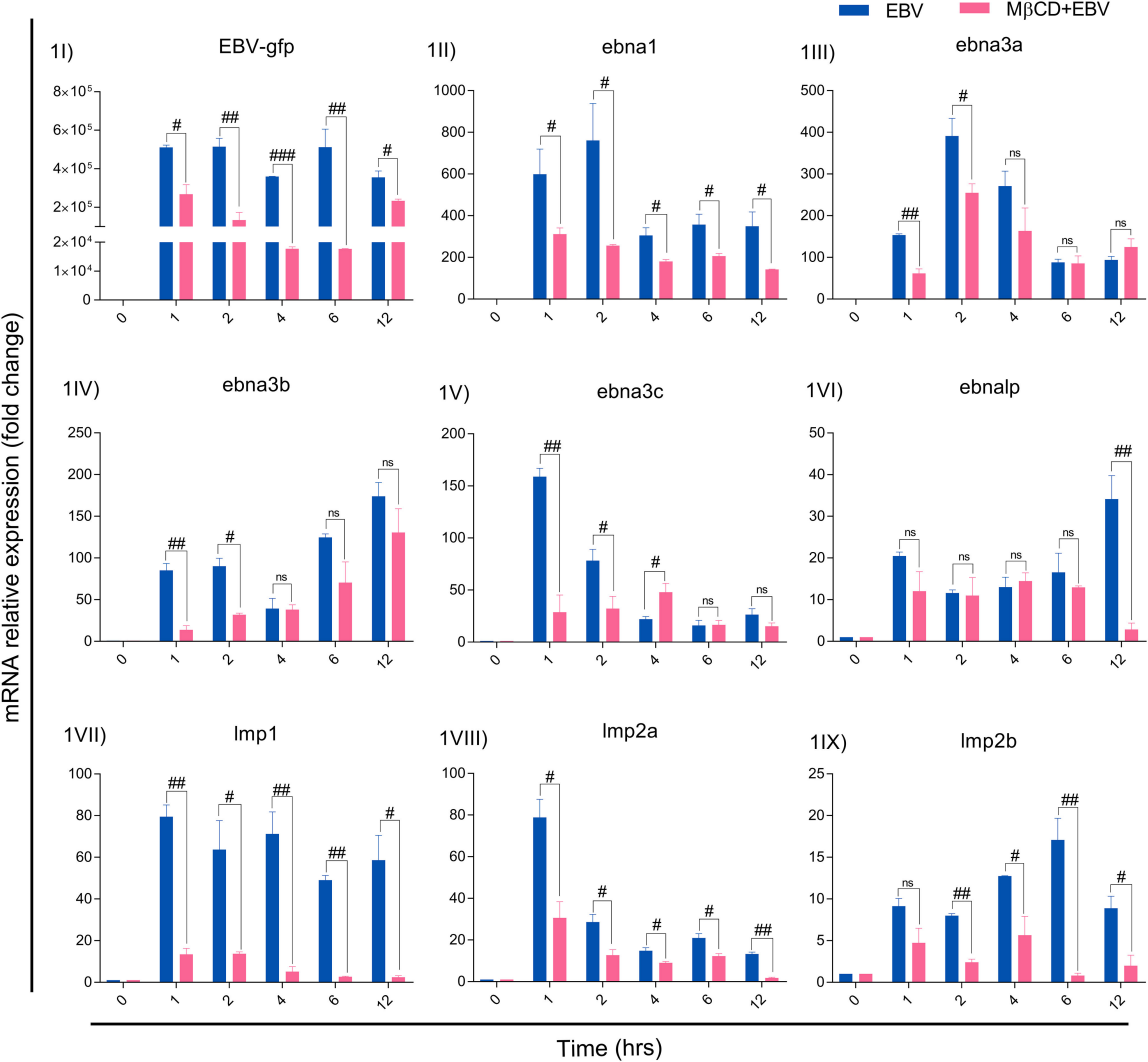
MβCD treatment curtails the expression of EBV latent genes in astroglia cells at different time points. **(I)** Reduced transcription of EBV-gfp, **(II)** ebna1, **(III)** ebna3a, **(IV)** ebna3b, **(V)** ebna3c and **(VI)** ebnalp manifested reduced transcription at different time points. Significant down-regulation of **(IV)** lmp1, **(VIII)** lmp2a and **(IX)** lmp2b expression in MβCD+EBV samples compared to EBV alone temporally. Given plots; *x*-axis, time-dependent EBV infection; *y*-axis, fold change with respect to EBV infected samples. The p-values of <0.05, <0.01 and p<0.001 are considered statistically significant and represented as #, ## and ###. Hereby, #/##/### represents the decrease in fold change, and ns represents the non-significant level. This experiment was performed on two biological and two technical replicates with glyceraldehyde 3-phosphate dehydrogenase (GAPDH) as a housekeeping gene.

For MβCD treatment of LN-229 cells, the IC_50_ value of MβCD was 3.008 mM. For further experiments, 1 mM concentration was used since more than 90% of cells were alive at this dose and subsequently used to infect with EBV (**Figure S1**). EBV titer was determined using qRT-PCR of EBV-green fluorescent protein (EBV-gfp) and EBV nuclear antigen 1 (ebna1) after infecting LN-229 cells at different concentrations (**Figure S2**). Therefore, 2.5 MOI was used to infect the cells as described previously (33). LN-229 cells were exposed to EBV alone and in the presence of MβCD for 1, 2, 4, 6 and 12 hrs and the mRNA levels of EBV latent genes were examined. Since the latent viral genes are critical to establishing a successful infection and/or maintaining latency, the mRNA levels of EBV-gfp, virus latent genes ebna1, -2, -3a, -3b, -3c, -lp, lmp1, -2a and -2b were checked. EBV-gfp showed a significant decrease in the presence of MβCD in comparison to EBV alone at 1, 2, 4, 6 and 12 hrs (p<0.05, p<0.01, p<0.001, p<0.01 and p<0.05 respectively) (**Figure 1I**). Likewise, the ebna1 transcript level get diminished significantly (p<0.01) in MβCD+EBV samples (**Figure 1II**). In contrast, ebna3a, -3b, and -3c and exhibited a significant decrease only in the initial hours (1, 2 and 4 hrs) post EBV infection while ebnalp indicated decline only at 12 hrs (p<0.01) (**Figures 1III-VI**). Interestingly, the lmp1 mRNA was significantly downregulated in MβCD+EBV samples compared to EBV infection alone at all time points 1, 2, 4, 6 and 12 hrs (p<0.01, p<0.05, p<0.01, p<0.01 and p<0.05 respectively) (**Figures 1VII–IX**). A similar pattern was observed for lmp2a and -2b transcripts as well (**Figures 1VIII, IX**).

### 3.2 EBV-mediated downstream signalling after membrane cholesterol disruption

**Figure 2:**
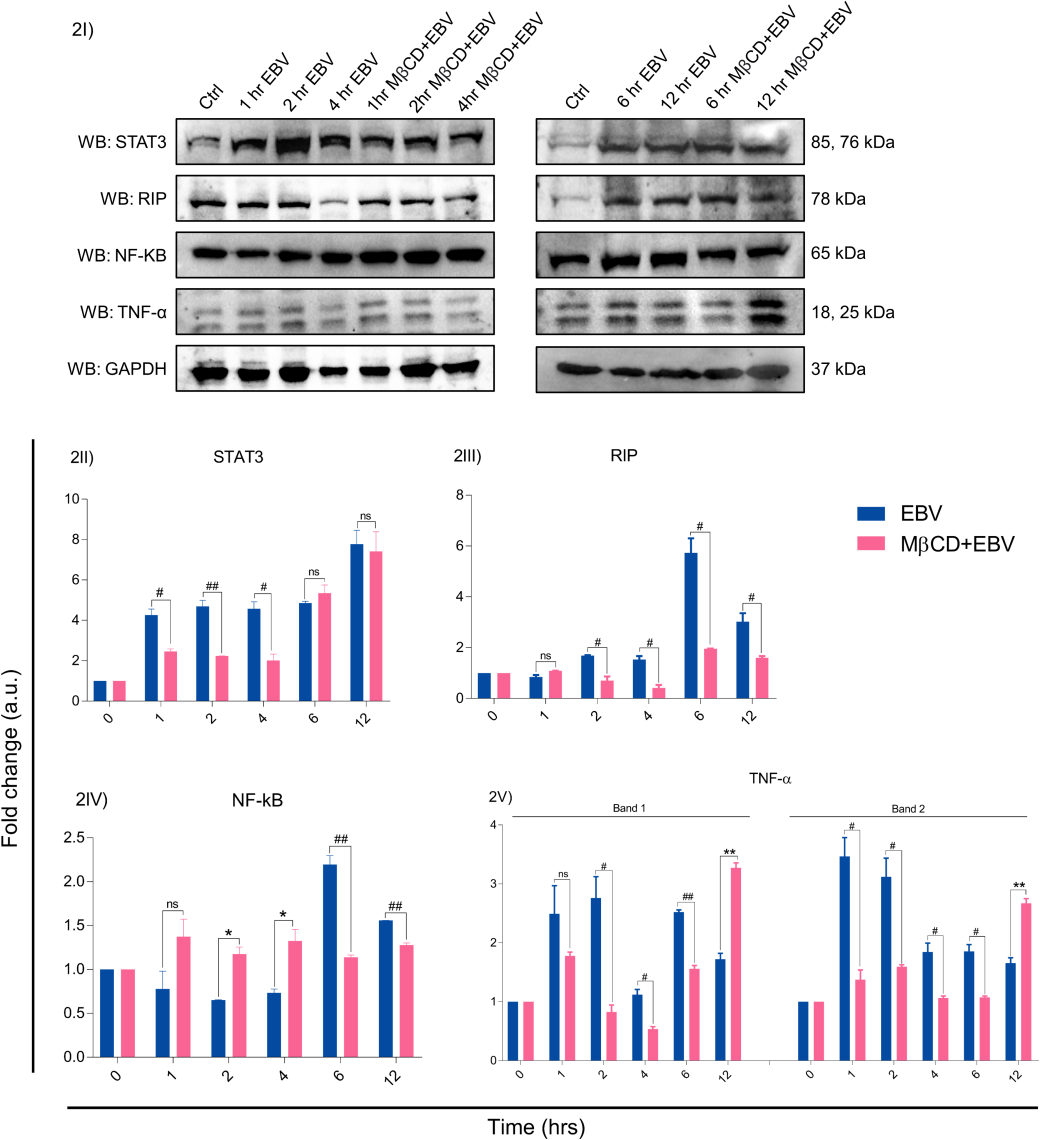
Representation of changes in EBV-mediated downstream signalling pathway proteins after MβCD treatment. **(I)** Western blot representation of STAT3, RIP, NF-kB and TNF-α. **(II-V)** Quantification of western blots. **(II)** STAT3 expression was reduced in MβCD treated samples at various time points i.e., 1, 2 and 4 hrs (p<0.05, p<0.01 and p<0.05 respectively). **(III)** RIP was also found to be decreased in MβCD+EBV compared to EBV-infected samples at 2, 4, 6 and 12 hrs (p<0.05). **(IV)** NF-kB was found to be increased after pre-treatment of MβCD at 2, 4 hrs and at 6, 12 hrs indicated decline. **(V)** TNF-α levels showed an initial decline upon exposure to MβCD at 2, 4 and 6 (p<0.05, p<0.05 and p<0.01) and at 12 hrs an increase in its expression was observed (p<0.01). Given plots; *x*-axis, time-dependent EBV infection; *y*-axis, fold change with respect to EBV infected samples. The p-values of <0.05 and <0.01 are considered statistically significant and represented as #/* and **/##. Hereby, */# represents the increase/decrease in fold change. The non-significant level is represented by ns. The increase and decrease are represented by * and # respectively. For the western blot glyceraldehyde 3-phosphate dehydrogenase (GAPDH) was used as a housekeeping gene and experiment was performed in two biological one technical repeats.

The EBV-encoded latent genes affect several critical downstream signalling pathways, we investigated protein levels of STAT3, RIP, NF-kB and TNF-α in LN-229 cells treated with MβCD+EBV and EBV alone at 0, 1, 2, 4, 6 and 12 hrs by immunoblotting (**Figures 2I**, **S5**). The results show that STAT3 exhibited decline in MβCD+EBV samples at 1, 2 and 4 hrs compared to EBV infected samples (p<0.05, p<0.01, p<0.05 respectively) except at 1 hr (**Figure 2II**). A similar pattern was observed for RIP kinase expression at 2, 4, 6 and 12 hrs (p<0.05) (**Figure 2III**). In contrast, the elevation in NF-kB was observed in MβCD+EBV samples compared to EBV alone at 2, and 4 hrs and at 6 and 12 hrs it manifested decline (p<0.01) (**Figure 2IV**). The expression of TNF-α was found to be decreased (both bands, p<0.05) in MβCD+EBV up until 6 hrs and the pattern was the opposite at 12 hrs of time point (**Figure 2V**).

### 3.3 Analysis of altered biomolecular fingerprint in EBV-infected astroglia cells after depleting the membrane cholesterol

#### 3.3.1 Raman spectral analysis

**Figure 3:**
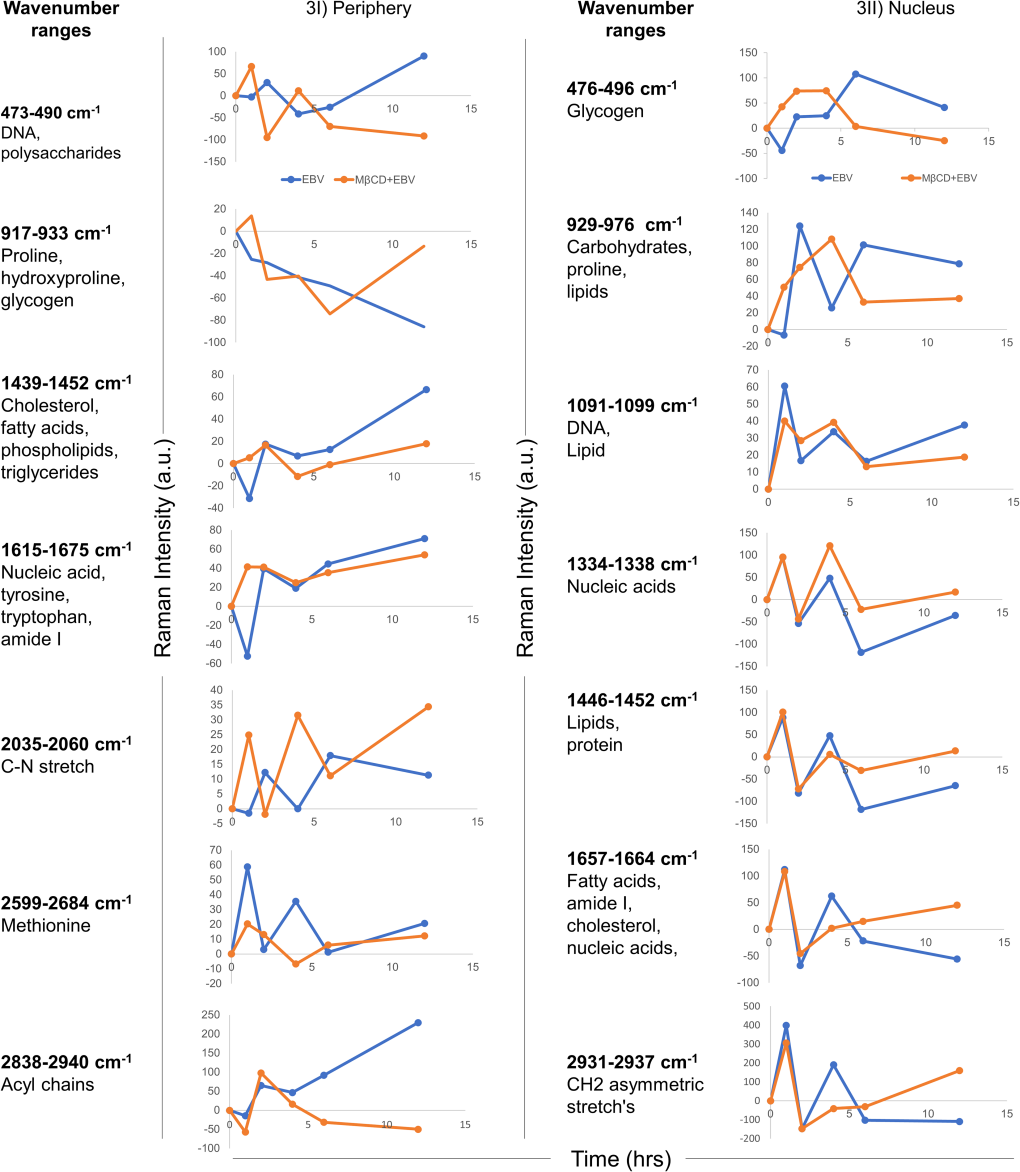
Temporal comparison of biomolecular signatures in EBV and MβCD+EBV exposed LN-229 cells. **(I)** Periphery, at 0, 1, 2, 4, 6 and 12 hrs. The wavenumber ranges represent specific cellular components- 476-496 cm-1 (glycogen), 929-976 cm-1 (carbohydrates and proline lipids), 1091-1099 cm-1 (DNA and lipid), 1334-1338 cm-1 (nucleic acids), 1446-1452 cm-1 (lipids and protein), 1657-1664 cm-1 (fatty acids, amide I, cholesterol and nucleic acids) and 2931-2937 cm-1 (CH2 asymmetric stretch’s). **(II)** Nucleus, a total of seven wavenumber ranges were observed which correspond to specific biomolecules. Specifically, these peak ranges from 473-490 cm-1 (DNA and polysaccharides), 917-933 cm-1 (proline, hydroxyproline and glycogen), 1439-1452 cm-1 (cholesterol, fatty acids and phospholipids), 1615-1675 cm-1 (nucleic acid, tyrosine, tryptophane and amide I), 2035-2060 cm-1 (C-N stretch), 2599-2684 cm-1 (Methionine) and 2838-2934 cm-1 (Acyl chains). The data were plotted as average spectra of 9 different points of three different cells.

To define the biomolecular fingerprints of EBV infection in astroglia cells and the role of cholesterol in it, we have used the Raman spectral analysis. We observed various Raman spectra in LN-229 cells exposed to EBV and MβCD+EBV samples at two different cellular locations namely, nucleus and periphery (transmembrane and cytoplasmic conjunction region of the cells). The raw data points of the spectra were plotted (wavenumber *vs* intensity) at 0, 1, 2, 4, 6, and 12 hrs (**Figure S3**). A total of seven major Raman peaks were observed at the periphery in the wavenumber ranges periphery 473-490, 917-933, 1439-1452, 1615-1675, 2035-2060, 2599-2684, and 2838-2940 cm^-1^ (**Figure 3I**). In periphery, the level of biomolecules like DNA and polysaccharides showed an increase in the MβCD+EBV samples at initial time points and eventually a decline was observed compared to only EBV exposed samples (**Figure 3I**). The level of cholesterol, fatty acids, phospholipids and triglycerides exhibited elevation in both EBV and MβCD+EBV samples initially and later there is abundant increase in the EBV-exposed samples (**Figure 3I**). Contrarily, nucleic acids, tyrosine, tryptophan, C-N stretch and methionine exhibited similar kind of changes in both type of samples (**Figure 3I**). Likewise, there were seven prominent peaks observed in the nucleus named as 476-496, 929-976, 1091-1099, 1334-1338, 1446-1452, 1657-1664 and 2931-2937 cm^-1^ (**Figure 3II**). Fatty acid, cholesterol, lipids and nucleic acids indicated decrease in only EBV infected samples compared to MβCD+EBV in the later time points (**Figure 3II**). While, the carbohydrates, glycogen was higher in EBV-exposed samples suggesting increase in the carbohydrate metabolism (**Figure 3II**).

#### 3.3.2 Raman intensity analysis of EBV-exposed LN-229 cells and its comparison with MβCD+EBV treated counterparts

**Figure 4:**
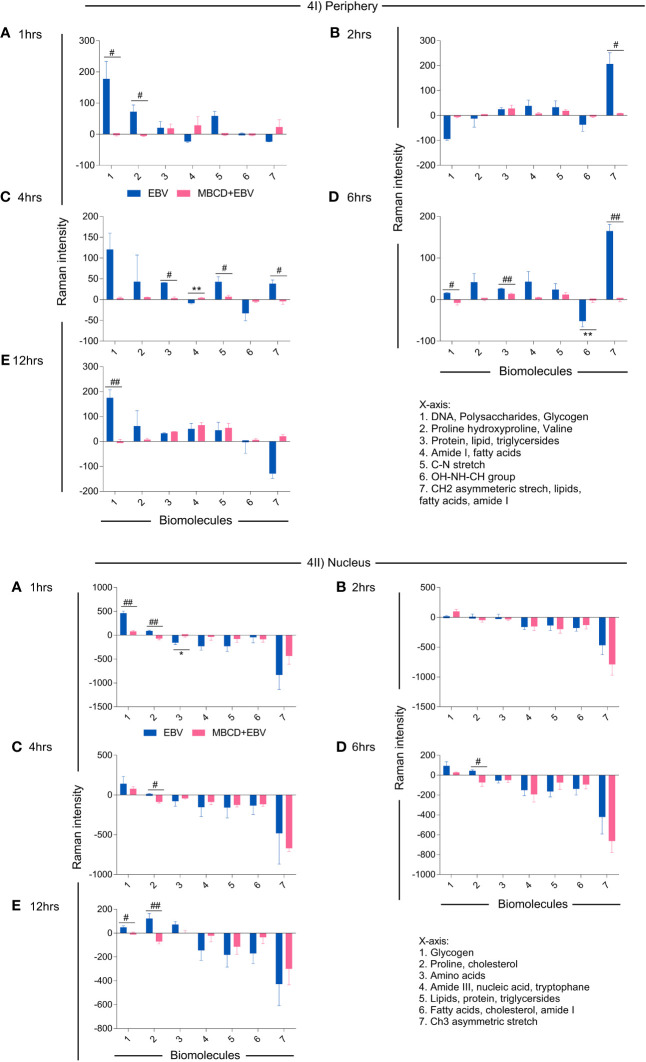
Illustration of changes in the biomolecular profile of LN-229 astroglia cells (periphery and nucleus) upon exposure to EBV and MβCD+EBV. **(I)** Altered biomolecules in the periphery (a) Augmented biomolecules in EBV-infected samples, (b) Increased molecules in LN-229 cells upon MβCD+EBV, (c) Declined biomolecules in EBV-infected samples, (d) Downregulated molecule in MβCD+EBV samples. **(II)** List of amended biomolecular signatures at the nucleus (a) Elevated biomolecules in EBV-infected samples, (b) Upregulated molecules in MβCD+EBV, (c) Downregulated molecules in EBV samples, (d) Reduced biomolecule profile in MβCD+EBV samples. The data were plotted as average spectra of nine points of three different cells. The p-values of <0.05 and <0.01 are considered statistically significant and represented as */# and **/##. Hereby, */# represents the increase/decrease in fold change. The non-significant level is represented as ns.

To investigate the spectral information corresponding to the biomolecular status, the signature spectra of unique biomolecules were listed (36–38), All the peak intensities were normalised with uninfected controls. The positive and negative variation, i.e., up and downregulation of intensity from the basal level of uninfected cells were further corroborated with the anabolic and catabolic activity of the molecules (**Figure 4**). In the periphery, biomolecule like DNA, polysaccharides, glycogen, proline, hydroxyproline and valine exhibited significant decline in MβCD+EBV samples at 1 hr of time point (p<0.05) (**Figure 4Ia**). Similarly, at 2 hrs CH_2_ asymmetric stretch and lipids showed a decrease (p<0.05) (**Figure 4Ib**). Further at 4 hrs lipids, triglycerides, C-N stretch and CH_2_ asymmetric stretch exhibited decline in MβCD+EBV samples compared to EBV infection (p<0.05) (**Figure 4Ic**). DNA and glycogen manifested significant decline after disruption of cholesterol at 6 and 12 hrs of time points (p<0.05, p<0.01 respectively) (**Figures 4Id, e**). In contrast, molecules like amide I, fatty acids and OH-NH-CH group indicated increase in MβCD+EBV samples at 4 and 6 hrs of time points respectively (p<0.01) (**Figures 4Ic, d**). Furthermore, in the nucleus, proline and cholesterol exhibited significant diminish at 1 (p<0.01), 4 (p<0.05), 6 (p<0.05) and 12 hrs (p<0.05) while protein showed significant upregulation in MβCD+EBV samples (p<0.05) (**Figures 4IIa–e**). Likewise, glycogen indicated significant decrease at 1 and 12 hrs of MβCD+EBV samples compared to EBV infection (p<0.01 and p<0.05 respectively) (**Figures 4IIa, e**).

#### 3.3.3 Analysis of biomolecular amendments using spectral width after depleting membrane cholesterol in astroglia cells

**Figure 5:**
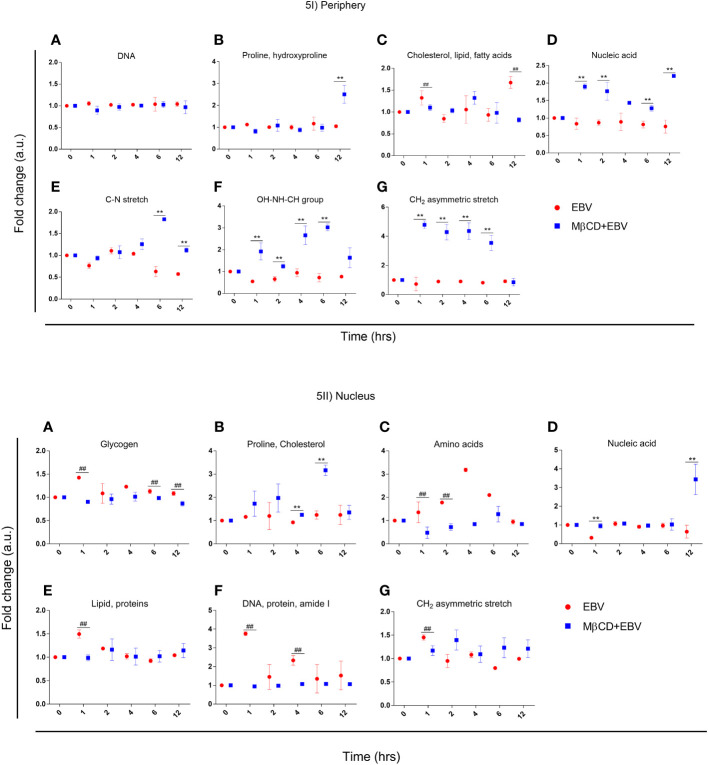
Representation of full-width half maxima of astroglia cells exposed to EBV and MβCD+EBV at periphery and nucleus. **(I)** FWHM of different biomolecules observed in the periphery. (a) Glycogen, (b) Proline and cholesterol, (c) Amino acids, (d) Nucleic acids, (e) Lipid and proteins, (f) DNA, protein and amide I, (g) CH2 asymmetric stretch. **(II)** FWHM of biomolecular alterations at the nucleus. (a) DNA (b) Proline and hydroxyproline, (c) Cholesterol, lipid and fatty acids, (d) Nucleic acid, (e) C-N stretch, (f) OH-NH-CH group, (g) CH2 asymmetric stretch. The data was represented as Mean ± SD of nine points of three different cells. The p-values of <0.05 and <0.01 are considered statistically significant and represented as */# and **/##. Hereby, */# represents the increase/decrease in fold change.

To investigate, biomolecular amendments after inhibition of cholesterol in EBV-infected astroglia cells and MβCD+EBV samples, we employed spectral width measurements of Raman peaks. At both cellular locations, i.e., periphery and nucleus, the full-width half maxima (FWHM) were calculated and normalized with uninfected control. FWHM represents the peak width of different biomolecules (35). A steeper peak width is related to the presence of unique biomolecules and a broader peak represents the presence of a derivative of that unique biomolecule (35). The FWHM was compared for samples with EBV alone and MβCD+EBV treatments (**Figure 5Ia**). At the periphery, the MβCD+EBV samples showed a significant elevation (p<0.01) in the proline, and hydroxyproline levels compared to EBV alone at 12 hrs of time point (**Figure 5Ib**). Interestingly, cholesterol, lipid and fatty acids exhibited a significant decline in the MβCD+EBV-exposed samples at 1 and 12 hrs (p<0.01) compared to MβCD+EBV samples (**Figure 5Ic**). While nucleic acid, OH-NH-CH group and CH_2_ asymmetric stretch significant increase at all times in the MβCD+EBV samples (p<0.01) (**Figures 5Id, If, Ig**). The C-N stretch depicted major augmentation in EBV samples at 6 and 12 hrs (p<0.01) (**Figure 5Ie**).

In nucleus, the FWHM related to proline and cholesterol was significantly up in MβCD+EBV (p<0.01) at 4 and 6 hrs of time point (**Figure 5IIb**). Molecules like glycogen, amino acids, protein, lipids and CH_2_ asymmetric stretch exhibited a decline in the initial time points (**Figures 5IIa, IIc, IIe, IIg**). FWHM of nucleic acid showed an increase in MβCD+EBV samples at 1 and 12 hrs compared to EBV infection (p<0.01) (**Figure 5IId**).

#### 3.3.4 LN-229 peripheral Raman peak shift in MβCD+EBV samples compared to EBV infection

**Figure 6:**
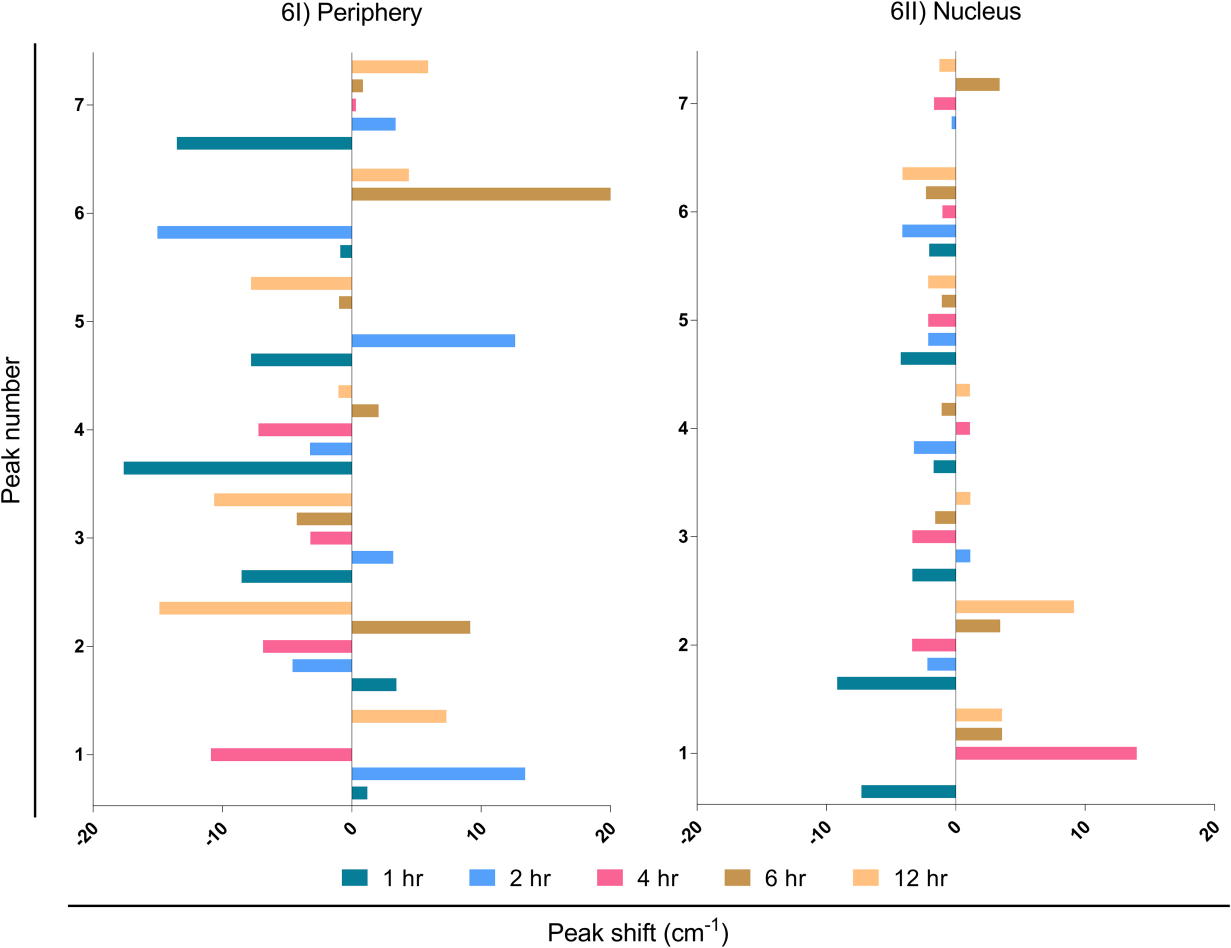
Interpretation of peak shift in Raman spectra from EBV to MβCD+EBV samples in the periphery and nucleus. **(I)** Peak shifts in the nucleus, **(II)** Peak shifts in the periphery. The data were plotted as average spectra of nine points of three different cells.

It is known that external stimuli including pathogens tend to trigger shifts in Raman peaks. Each peak corresponds to a specific molecular bond vibration (39). Shifts in these peaks at lower (blue shift) and higher (red shift) wavenumbers are due to the chemical bond length of molecules (40). The larger bond length causes a shift to a lower wavenumber and vice-versa (41). Major peak shifts were observed in LN-229 cells at the periphery compared to the nucleus (**Figure 6I**; **Table S2**). The peak shift data was observed by subtracting the wavenumber maxima of EBV samples from MβCD+EBV and ±5 cm^-1^ peak shift was considered for further biomolecular signature observation (**Table S2**). The first peak shift was observed in the periphery at 2, 4 and 12 hrs (**Figure 6I**). At 2 and 4 hrs, the Raman shift corresponds to the same molecule, namely carbohydrate. In comparison, the shift at 12 hrs suggests conversion of polysaccharides into DNA (**Figure 6I**). At 12 hrs, the second peak indicated a major change from carbohydrates to RNA (**Figure 6I**). Importantly, the third peak at 1hr manifested a change from protein band to triglycerides and lipids. Notably, the fourth peak showed major transpose at 1 and 4 hrs, whereas at 1 hr it exhibited a swap of amide I into tyrosine and tryptophan (**Figure 6I**). While, at 4 hrs protein got changed with amide I. No major changes were observed in peak number six and seven (**Figure 6I**). No considerable peak shifts were observed in the nucleus except at 1 and 2 hrs which showed shifts into the same biomolecule such as glycogen to glycogen and polysaccharides to glycogen (**Figure 6II**).

#### 3.3.5 Ingenuity pathway analysis of altered biomolecular signature in LN-229 cells

**Figure 7:**
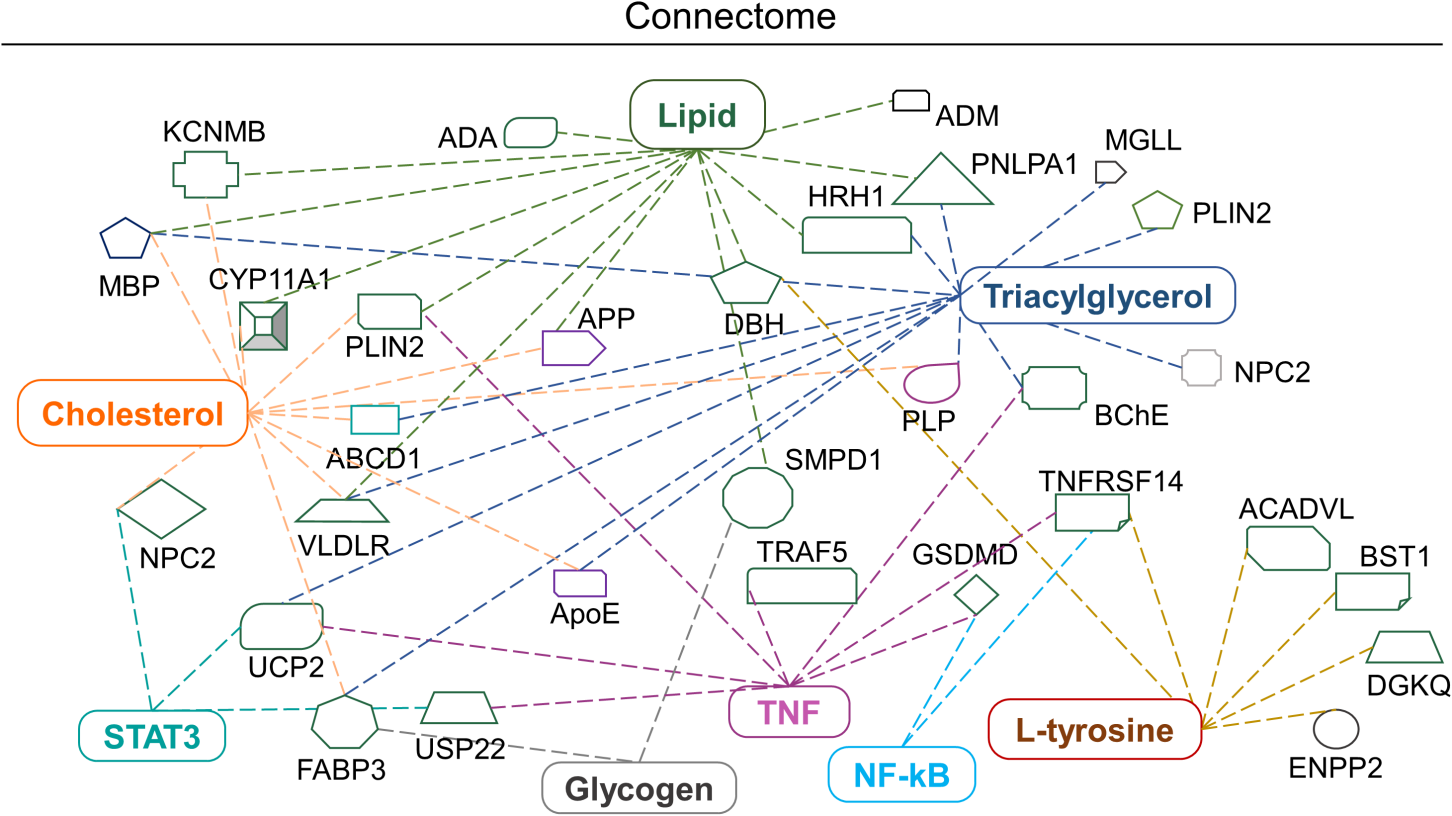
Biomolecular connectome of astroglia cells by IPA. The connectome shows the factors involved in infectious, neurological and inflammatory diseases and responses as revealed by the IPA knowledge database. A majority of the biomolecules observed in the astroglia cells after EBV infection in the intact and disrupted membrane cholesterol linked to various individual or familiar molecular entities are also present in the network.

IPA tools were used to identify the possible biomolecule-associated pathways and gene networks that correspond to infectious diseases, inflammatory responses and neurological disorders in the CNS and neuronal cell lines. The comprehensive molecular network, ingenuity pathway knowledge base (IPKB) identified canonical/non-canonical pathways and gene networks associated with specific biomolecule-related neuropathologies. The prominent altered molecules obtained after EBV infection in cholesterol-depleted and intact astroglial cells include cholesterol, triglycerides, lipids, glycogen and aromatic amino acids. Likewise, the biomolecules subjected to IPA input were cholesterol, triacylglycerol, L-tryptophane, L-tyrosine, L-proline, hydroxyproline, lipids, glycogen and proteins like RIP, STAT, TNF and NF-kB (**Figure 7**). Intriguingly, cholesterol trafficking is acclaimed to be altered in several neurological disorders (42).

Briefly, the IPA connectome unravelled new proteins associated with altered cholesterol and lipid such as Neimann-Pick C2 (NPC2), perilipin 2 (PLIN2), histamine receptor H1 (HRH1) and very low-density lipoprotein receptor (VLDLR) and patatin-like phospholipase domain containing 1 (PNPLA1). Similarly, mitochondrial uncoupling protein 2 (UCP-2) declines ATP production and expression in the brain and reduces oxidative stress (**Figure 7**). Altered L-tyrosine, triglycerides and glycogen showed association with enzymes like dopamine beta-hydroxylase (DBH) which play a role in the conversion of dopamine into nor-epinephrine (**Figure 7**). While butyrylcholinesterase (BChE) plays an important role in the production of pseudocholinesterase (**Figure 7**). Amendments in the lipid profile are connected with potassium calcium-activated channels subfamily M regulatory bet subunit 1 (KCNMB1) and adrenomedullin (ADM) proteins which are widely expressed in the brain (**Figure 7**). Triglycerides, lipids and cholesterol showed an interrelation with the cholesterol side-chain cleavage enzyme (CYP11A1) (**Figure 7**). Glycogen and cholesterol changes draw a link with fatty acid binding protein 3 (FABP3) (**Figure 7**). The networking pathway of glycogen and lipid revealed the relation with sphingomyelin phosphodiesterase 1 (SMPD1), acyl-CoA dehydrogenase very long chain (ACADVL) (**Figure 7**).

## 4 Discussion

Given the interesting association of EBV with neurological diseases has been corroborated by an increasing number of studies, yet the mechanistic details are still ambiguous (4, 7, 43), Previous reports suggested that in case of EBV entry, cholesterol plays an important role in membrane fusion, receptor localization in membrane microdomains and/or early viral signalling events or fusion of the EBV envelope with the cellular membrane might require a cholesterol-rich environment (44). Moreover, cholesterol and lipid moieties are found in abundance within the brain. The largest pool of free cholesterol in the myelin sheath is a crucial component taking part in electrochemical conduction along the axons. Therefore, disruption in cholesterol homeostasis becomes detrimental, as seen in MS at its various stages. The present investigation sought to understand the role of membrane cholesterol in EBV-mediated pathogenesis. By using MβCD, we noted a decline in the expression of EBV-gfp and ebna1 which suggest a delay in EBV infection. A similar result was observed earlier after depleting the cholesterol in order to understand EBV entry into Daudi B-cell (14). Also, cholesterol depletion in LCL cells exhibited a blockage in the LMP2A endocytosis resulting in its accumulation on plasma membrane (14, 16). Notably, our results also showed downregulation of lmp1 and lmp2a upon disruption of membrane cholesterol, underpinning its important role in reducing EBV entry/viral gene expression into astroglia cells. Interestingly, an *in-vivo* study suggested that MBP-specific antibodies exhibited cross-reactivity with EBV-LMP1 and insisted its role in MS pathology (45). Besides, the effect on downstream signalling molecules affected by EBV infection in cholesterol-depleted astroglia cells was examined by analysing STAT3, RIP, NF-kB and TNF-α expression at protein level. EBV infection triggers the activation of STAT3 and RIP proteins and contributes to neuroinflammation and cell death (46). In our study, inhibition of cholesterol led to reduced expression of the above proteins in astroglia cells except for NF-kB. Likewise, a study by Vorst et al. suggested, the depletion of membrane cholesterol leads to increased expression of NF-kB (47). Notably, EBV-LMP1 has been found to constitutively activate NF-kB through numerous mechanisms and instigates the cytokine storm in case of autoimmune disease like MS (**Figure S4**). Several MS therapeutics drugs like fingolimod have been implicated as an influencer of NF-kB signalling (48). EBNA1-specific CD4^+^ T-cell clones cross-react with peptide mixture derived from CNS autoantigens. These autoantigens are capable of producing IL-2, IFN-γ and TNF-α cytokines and imparts in MS pathology (49). This is in line with data by others, which showed that disrupted membrane cholesterol in Raji B-cells blocked the cytotoxicity of transmembrane TNF (50). The IPA network suggested a direct link of RIP and TNF with caspase recruitment protein with FGF-like domain 1 (CARD6) and TNF Receptor Associated Factor 5 (TRAF5) (**Figures 7**, **S4**). In order to explore the detail biomolecular changes in the current infection model, RS was used for checking the systemic biomolecular signature.

Precisely, the cholesterol and related moieties showed major changes in EBV-infected samples at the cell periphery while this pattern was reversed at the initial time points of nucleus. MβCD removes cholesterol molecules from the cell membrane, thus MβCD+EBV samples showed a low presence of cholesterol and related molecules in the periphery. While in the nucleus of MβCD+EBV samples, cholesterol, triglycerides and lipids continued to show their prominent presence. The accumulation of cholesteryl ester in the brain is scrutinized to be linked with demyelination during MS and other CNS demyelinating diseases (51). It suggests that the differential cellular changes in the nucleus and periphery are due to maintenance of cellular homeostasis. In the cellular periphery, EBV infection showed an increase in Raman intensity of triglycerides and fatty acids which were not observed after membrane cholesterol turmoiling. The aforementioned alterations are due to EBV-mediated changes in the cells and suggesting a reduction in viral entry and consequent pathogenesis such as MS.

The energy shifts of RS can be used to obtain information regarding the molecular composition of the sample with very high accuracy (52). In the current samples, the periphery of the cells showed more shifts compared to the nucleus at initial time points. A shift at the periphery indicated a change from protein to triglycerides and lipids. These molecules have a pleiotropic role in viral infection (53–55). The shift in these molecules also suggested an increase in their number which may help in maintaining the membrane fluidity. Previously, severe hypertriglyceridemia concentrations >300 mg/dL was been reported during infectious mononucleosis (56). Patients with infectious mononucleosis showed a higher incidence of MS than to patients without infectious mononucleosis (57). Albeit, the ingenuity connectome showed that lipids and cholesterol were found to be prominently linked with receptors like HRH1/VLDLR and altered function of these receptors has a significant role in infection-related neuroinflammation (**Figure 7**). Likewise, compared to healthy subjects, in the MS patients had elevated sub-fractions of VLDL and HDL lipoprotein (58). Furthermore, Raman shifts analysis indicated two major transposes including polysaccharides to DNA and carbohydrates to RNA. These data are pointing towards an increase in replication and transcription. We observed an elevation in the triglycerides and fatty acids upon exposure to EBV at the periphery whereas it was absent in the cholesterol-depleted LN-229 cells. Alterations in the lipid profile seem to be a hallmark of this pathology which can contribute to the dysregulation of lipid homeostasis and metabolism in MS (59). The clinical lipidomic profile is potent as a tool to aid in MS diagnosis and therapeutics by allowing a detailed lipidome profiling of the patients suffering with disease (60). The connectome of triglycerides highlighted a consortium with UCP-2 and CYP11A1. Our data further show how usage of MβCD can decrease EBV gene expression and eventually reduce activation of CYP11A1, UCP-2 and related neuropathologies (**Figure 7**). The latter converts cholesterol into pregnenolone and serves as a substrate for vitamin D2/D3 (**Figure 7**). Vitamin D deficiency is also a diverse risk factor for MS. Notably, 1,25(OH)2D3 and vitamin D receptor were shown to interact with EBNA1 and contribute to MS. EBNA2 and VDR have common DNA binding sites associated with MS (61).

Previous studies have suggested that the amide I signature represents sphingomyelin which is widely present in the plasma membrane (62, 63). Likewise, we observed peak width of amide I showed an increase in the EBV-infected cells in the periphery at the initial time points. Additionally, elevated Raman spectra for the Phe-Tyr ratio have been used to measure phenylalanine hydroxylase activity that is indirectly related to immune activation and inflammatory process through tetrahydrobiopterin (BH4) (64). The MβCD treated samples showed an elevation in tyrosine and tryptophane which indicates that EBV potentially alters these amino acids immediately after the infection to circumvent immune responses. Altogether, our results highlight the important role of membrane cholesterol in EBV entry/pathogenesis in astroglia cells which might further trigger or exacerbate virus-associated neuropathologies.

## 5 Conclusion

Briefly, to the best of our knowledge, the present study shows for the first time how membrane cholesterol plays a critical role in EBV infection of astroglia cells. We also discerned molecular pathways affected by cholesterol depletion. Using the sophisticated RS technology, we have identified the molecular fingerprint of EBV and its interaction with astrocytes. In view of the recent and growing evidence of EBV’s involvement in MS and other neuropathologies, our data set the stage for a better understanding how EBV may contribute to neurological pathologies

## Data availability statement

The original contributions presented in the study are included in the article/**Supplementary Material**. Further inquiries can be directed to the corresponding authors.

## Ethics statement

Ethical approval was not required for the studies on humans in accordance with the local legislation and institutional requirements because only commercially available established cell lines were used.

## Author contributions

AR: Conceptualization, data curation, original draft writing, figure preparation. MT and PP helped in critical review and editing. TV helped in critical review, figure/table preparation and editing. PT and RK Critical review and editing. HJ: Conceptualization, critical review and editing. All authors contributed to this article and approved the submitted version.
