# Supplementary material for: Understanding the role of membrane cholesterol upon Epstein Barr virus infection in astroglial cells
Source: Front Immunol. 2023 Oct 9;14:1192032. doi: 10.3389/fimmu.2023.1192032 (PMC10591182; doi:10.3389/fimmu.2023.1192032)
Supplement: Supplementary file 1 [file DataSheet_1.docx]

**Supporting information**

**Understanding the role of membrane cholesterol upon Epstein Barr virus infection in astroglial cells**

Authors: Annu Rani^1^, Manushree Tanwar^2,3^ Tarun Prakash Verma1^#^, Priyanka Patra1^#^, Pankaj Trivedi^4^, Rajesh Kumar^2*^, Hem Chandra Jha^1*^

^1^Department of Biosciences and Biomedical Engineering, Indian Institute of Technology Indore, MP, India.

^2^Materials and Device Laboratory, Department of Physics, Indian Institute of Technology Indore, MP, India

^3^Department of Chemistry, University of Pennsylvania, Philadelphia, Pennsylvania 19104-6323, United States

^4^Department of Experimental Medicine, Sapienza University of Rome, Viale Regina Elena 324, 00161 Rome, Italy

^#^ Equal contribution

^*^ Corresponding Author

Address for correspondence:

Dr Hem Chandra Jha, Infection Bioengineering Group, POD 1B-602, Department of Biosciences and Biomedical Engineering, Indian Institute of Technology, Indore, Madhya Pradesh, PIN 453552, Office: 0731-4306581, Email ID: hemcjha@iiti.ac.in, Phone: +91-9971653189

Dr Rajesh Kumar, Materials and Device Laboratory, Discipline of Physics, Indian Institute of Technology Indore, MP, India. Email ID: rajeshkumar@iiti.ac.in


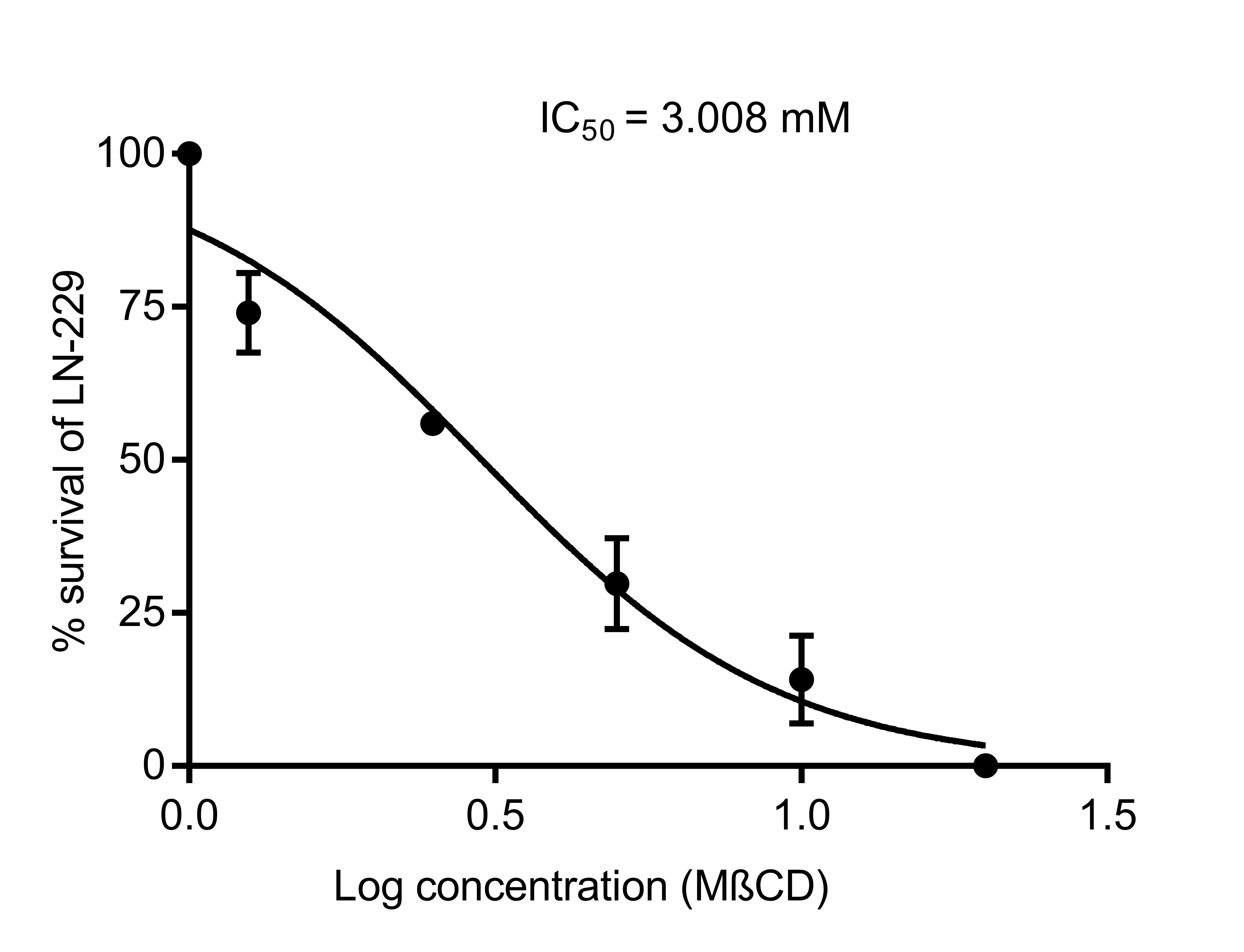


**Fig. S1:** Cell cytotoxicity assay of LN-229 cells upon MβCD treatment. Inhibitory concentration 50 (IC_50_) was 3.008 mM.


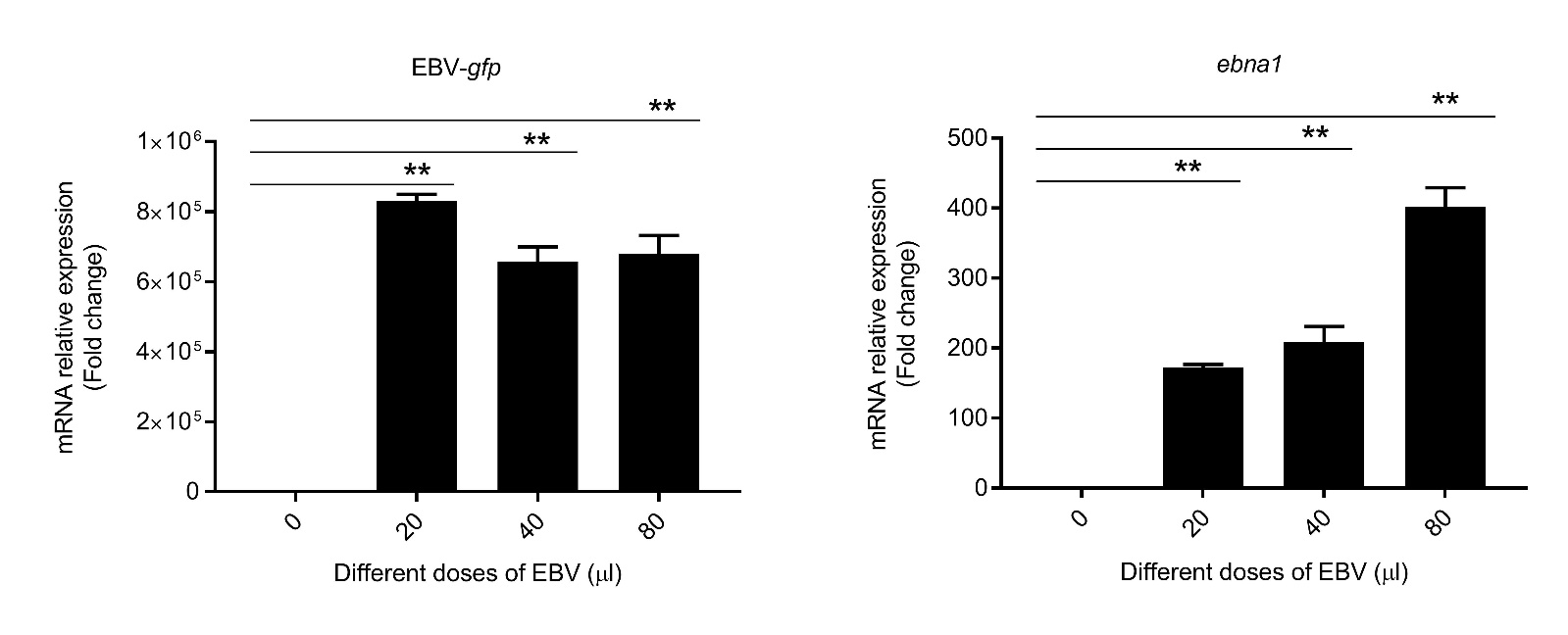


**Fig. S2:** EBV titer determination on LN-229 cells through qRT-PCR. From this graph the 25 μl of diluted EBV corresponds to 2.5 MOI.


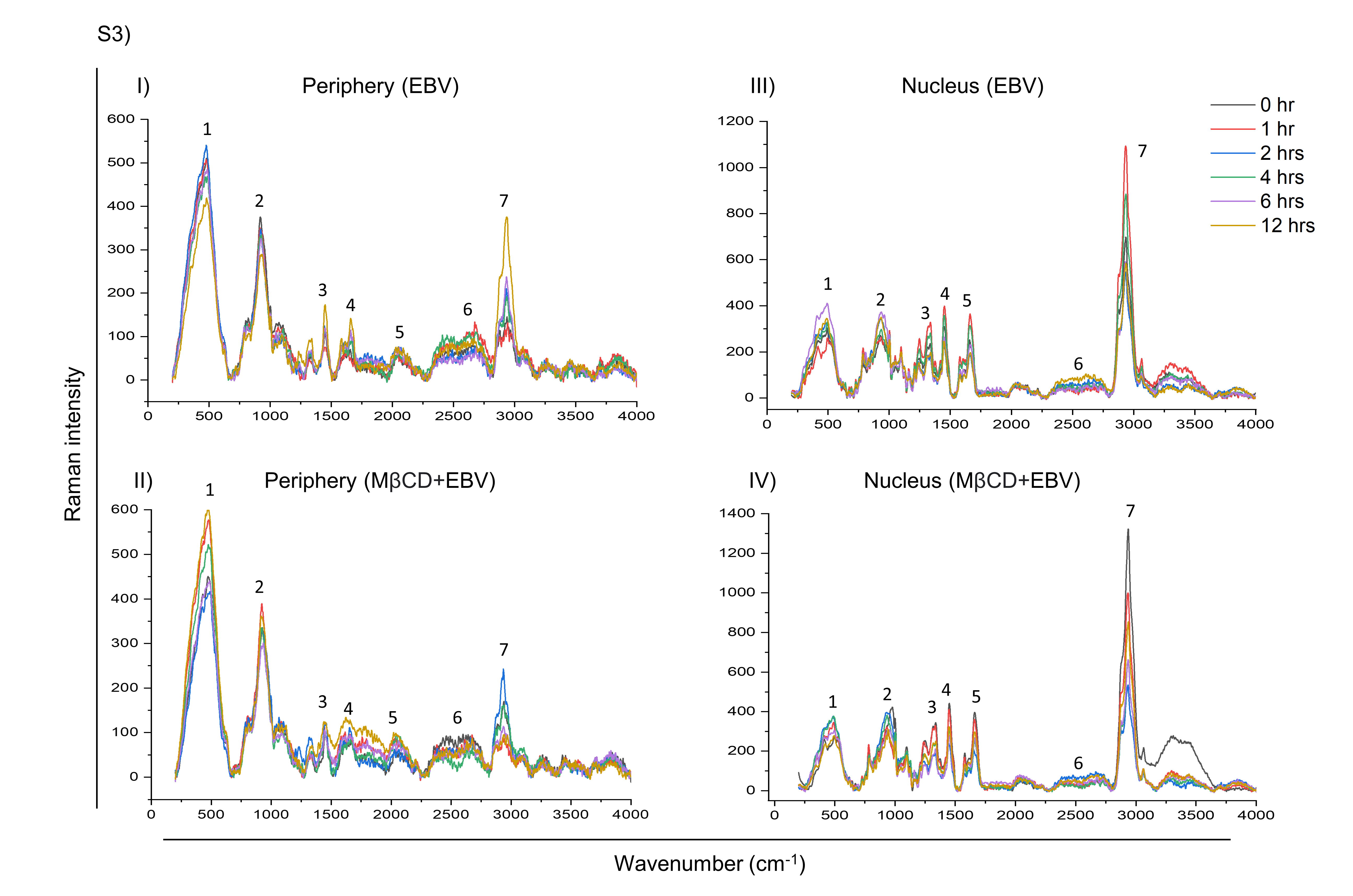


**Fig. S3:** Raman spectra in EBV and MβCD+EBV exposed LN-229 astroglial cells. I) Raman spectra of the periphery for only EBV exposed sample, II) Periphery spectra for MβCD+EBV cells, III) Spectra for nucleus after EBV infection and IV) Spectra for nucleus for MβCD+EBV samples. The data were plotted as average spectra of 9 different points of three different cells.


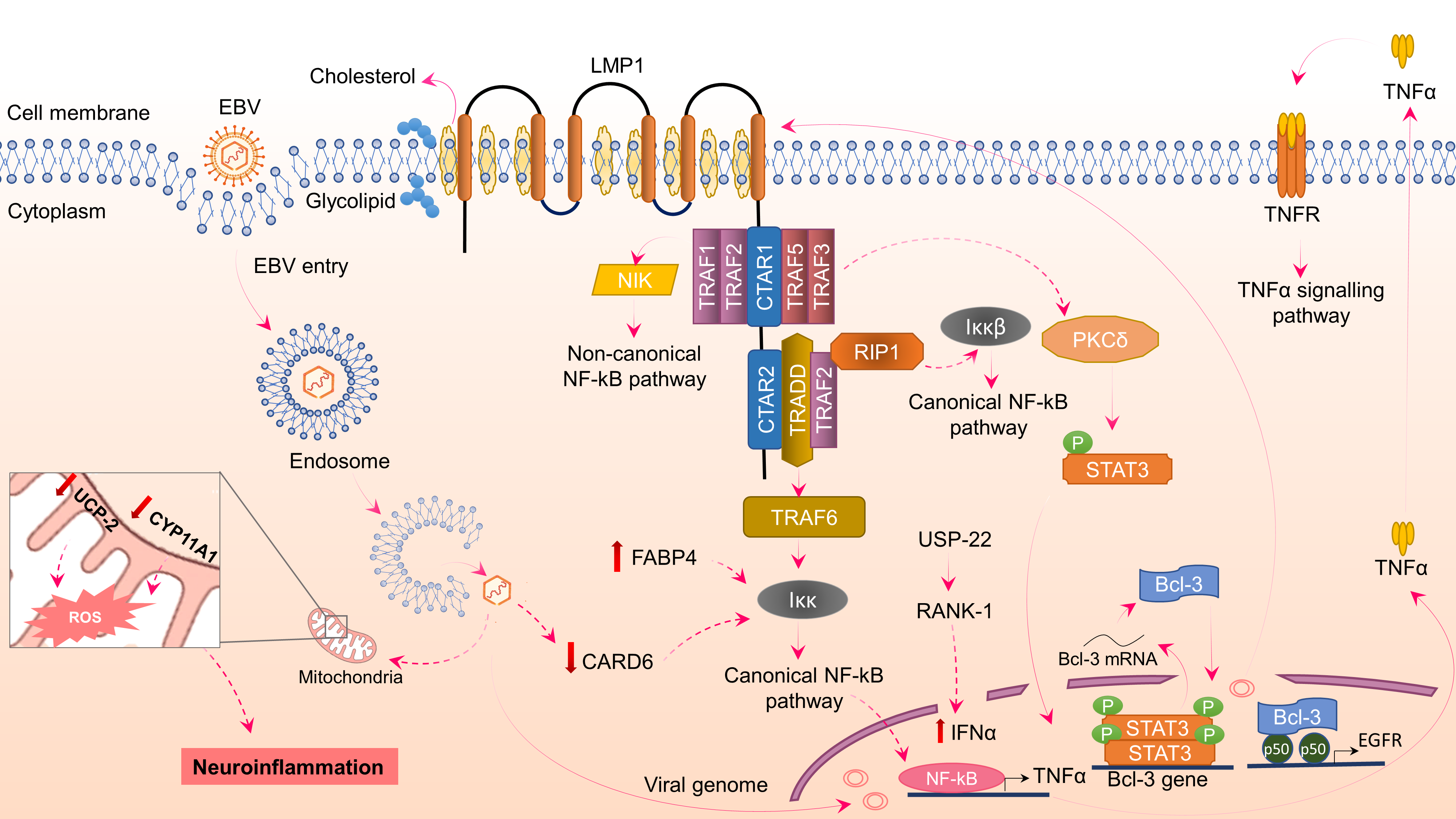


**Fig. S4:** A schematic representation of EBV downstream signalling cascade in astroglial cells. CTAR1 and CTAR2 domains of EBV-LMP1 interact with TRAFs which recruits further RIP kinase, NIK and STAT3. RIP in turn activates the canonical NF-kB pathway and NIK activates the non-canonical NF-kB pathway. Phosphorylated STAT3 form dimers. STAT3 dimers and NF-kB then migrate into the nucleus and up-regulate the expression of inflammatory cytokines. TNFα is one of the cytokines activated by NF-kB which is responsible for the expression of adhesion molecules over cells increasing the adhesion of leukocytes and contributing to ROS at the site of infection. TNFα increases its expression and contributes to cytokine storms. Increased level of FABP4 and USP-22 activates Iκκ and RANK-1 respectively. RANK-1 increase the level of IFNα. Decreased levels of CARD6 contribute to the activation of NF-kB canonical pathway. Abnormal expression of mitochondrial protein UCP-2 and CYP11A1 contribute to the release of ROS and cumulatively these responses contribute to neuroinflammation.

**Table S1:** List of qRT-PCR primers used in the study.

| **Sr. no** | **Genes** | **Primer Sequence** |
| --- | --- | --- |
| 1 | Glyceraldehyde-3-Phosphate Dehydrogenase (GAPDH) | F: TGCACCACCAACTGCTTAG |
|  |  | R: GATGCAGGGATGATGTTC |
| 2 | Green fluorescent protein (GFP) | F: ACGTAAACGGCCACAAGTTC |
|  |  | R: AAGTCGTGCTGCTTCATGTG |
| 3 | EBV nuclear antigen 1 (EBNA1) | F: CACCATTGAGTCGTCTCCCC |
|  |  | R: TCAAAGCTGCACACAGTCAC |
| 4 | EBV nuclear antigen 2 (EBNA2) | F: GAACTTCAACCCACACCATC |
|  |  | R: CGTGGTTCTGGACTATCTGG |
| 5 | EBV nuclear antigen 3A (EBNA3A) | F: GGTGAAACGCGAGAAGAAAG |
|  |  | R: TTTAGCAGTTCCTCCGCACT |
| 6 | EBV nuclear antigen 3B (EBNA3B) | F: AGAAGAGGCCCTTGTGTCTT |
|  |  | R: GGATTTCAAGAGGGTCAGGT |
| 7 | EBV nuclear antigen 3C (EBNA3C) | F: AGAAGGGGAGCGTGTGTTGT |
|  |  | R: GGCTGGTTTTTGACGTCGGC |
| 8 | EBV nuclear antigen LP (EBNALP) | F: TCCCCTCGGACAGCTCCTA |
|  |  | R: CCACTTACCACCTCCCCTTCT |
| 9 | Latent membrane protein 1 (LMP1) | F: CCCGCACCCTCAACAAGCTACCGAT |
|  |  | R: TTGTCAGGACCACCTCCAGGTGCGC |
| 10 | Latent membrane protein 2A (LMP2A) | F: CTACTCTCCACGGGATGACTCAT |
|  |  | R: GGCGGTCACAACGGTACTAACT |
| 11 | Latent membrane protein 2B (LMP2B) | F: CGGGAGGCCGTGCTTTAG |
|  |  | R: GGCGGTCACAACGGTACTAACT |

**Table S2:** Enlistment of wavenumber maxima for nucleus and periphery. Peak shift (Δcm^-1^) is indicating the difference in the peak maxima of MβCD+EBV and EBV samples.

|  | | Nucleus | | | | | Periphery | | | | |
| --- | --- | --- | --- | --- | --- | --- | --- | --- | --- | --- | --- |
| Time (hrs) | | 1 | 2 | 4 | 6 | 12 | 1 | 2 | 4 | 6 | 12 |
| Peak 1 | **EBV** | 496.41 | 487.89 | 476.40 | 491.54 | 491.54 | 475.72 | 476.93 | 486.67 | 483.03 | 473.28 |
|  | **MβCD+EBV** | 489.11 | 487.89 | 490.38 | 495.12 | 495.12 | 476.93 | 490.33 | 475.78 | 483.03 | 480.59 |
|  | **Peak shift (Δcm^-1^)** | -7.30 | 0.00 | 13.98 | 3.58 | 3.58 | 1.21 | 13.40 | -10.89 | 0.00 | 7.31 |
| Peak 2 | **EBV** | 941.57 | 931.20 | 935.86 | 933.58 | 933.58 | 917.56 | 921.00 | 927.86 | 924.43 | 931.29 |
|  | **MβCD+EBV** | 932.40 | 929.00 | 932.49 | 937.01 | 942.72 | 921.00 | 916.42 | 921.00 | 933.58 | 916.42 |
|  | **Peak shift (Δcm^-1^)** | -9.17 | -2.20 | -3.37 | 3.43 | 9.14 | 3.44 | -4.58 | -6.86 | 9.15 | -14.87 |
| Peak 3 | **EBV** | 1097.30 | 1091.72 | 1099.53 | 1097.30 | 1096.18 | 1452.25 | 1443.73 | 1446.93 | 1447.99 | 1450.12 |
|  | **MβCD+EBV** | 1093.95 | 1092.83 | 1096.18 | 1095.70 | 1097.30 | 1443.73 | 1446.93 | 1443.73 | 1443.73 | 1439.47 |
|  | **Peak shift (Δcm^-1^)** | -3.35 | 1.11 | -3.35 | -1.60 | 1.12 | -8.52 | 3.20 | -3.20 | -4.26 | -10.65 |
| Peak 4 | **EBV** | 1337.37 | 1337.37 | 1337.37 | 1336.29 | 1337.37 | 1632.82 | 1655.70 | 1667.94 | 1658.66 | 1658.66 |
|  | **MβCD+EBV** | 1335.65 | 1334.14 | 1338.45 | 1335.21 | 1338.45 | 1615.19 | 1652.47 | 1660.73 | 1660.73 | 1657.63 |
|  | **Peak shift (Δcm^-1^)** | -1.72 | -3.23 | 1.08 | -1.08 | 1.08 | -17.63 | -3.23 | -7.21 | 2.07 | -1.03 |
| Peak 5 | **EBV** | 1452.25 | 1449.06 | 1452.25 | 1451.19 | 1450.12 | 2043.27 | 2051.04 | 2043.27 | 2060.75 | 2045.22 |
|  | **MβCD+EBV** | 1447.99 | 1446.93 | 1450.12 | 1450.12 | 1447.99 | 2035.49 | 2063.66 | 2043.27 | 2059.78 | 2037.44 |
|  | **Peak shift (Δcm^-1^)** | -4.26 | -2.13 | -2.13 | -1.07 | -2.13 | -7.78 | 12.62 | 0.00 | -0.97 | -7.78 |
| Peak 6 | **EBV** | 1661.76 | 1657.63 | 1660.73 | 1662.00 | 1664.85 | 2674.66 | 2678.19 | 2630.34 | 2684.36 | 2669.36 |
|  | **MβCD+EBV** | 1659.70 | 1653.50 | 1659.70 | 1659.70 | 1660.73 | 2673.77 | 2663.17 | 2630.34 | 2780.83 | 2673.77 |
|  | **Peak shift (Δcm^-1^)** | -2.06 | -4.13 | -1.03 | -2.30 | -4.12 | -0.89 | -15.02 | 0.00 | 96.47 | 4.41 |
| Peak 7 | **EBV** | 2934.74 | 2936.50 | 2937.28 | 2931.36 | 2937.70 | 2943.19 | 2932.21 | 2937.80 | 2934.74 | 2935.59 |
|  | **MβCD+EBV** | 2934.74 | 2936.19 | 2935.59 | 2934.74 | 2936.43 | 2929.67 | 2935.59 | 2938.12 | 2935.59 | 2941.50 |
|  | **Peak shift (Δcm^-1^)** | 0.00 | -0.31 | -1.69 | 3.38 | -1.27 | -13.52 | 3.38 | 0.32 | 0.85 | 5.91 |

**
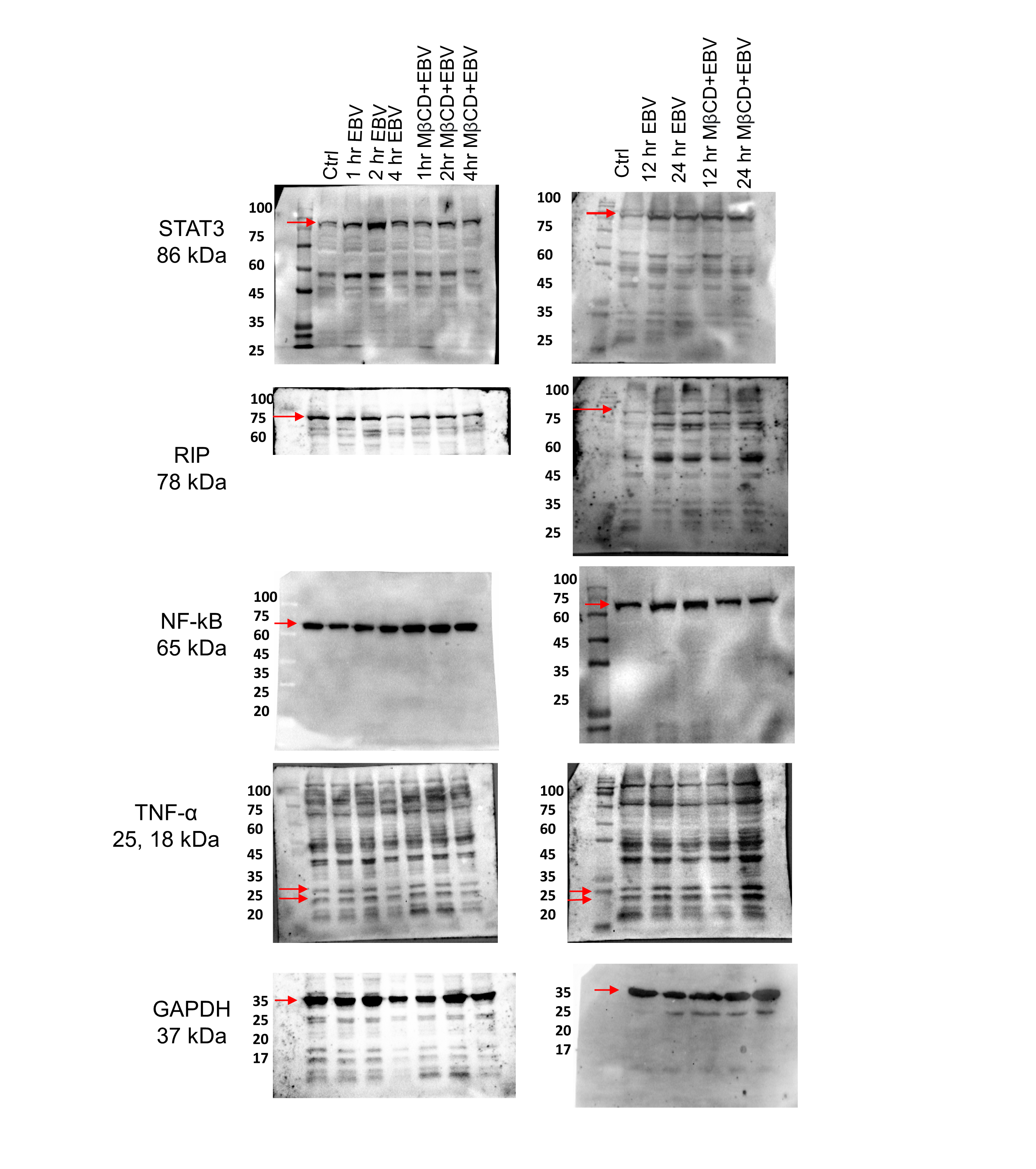
**

**Fig. S5:** Complete western blot image STAT3, RIP, NF-kB, TNF-α and GAPDH.
